# Effect and safety of treatment with ACE-inhibitor Enalapril and β-blocker metoprolol on the onset of left ventricular dysfunction in Duchenne muscular dystrophy - a randomized, double-blind, placebo-controlled trial

**DOI:** 10.1186/s13023-019-1066-9

**Published:** 2019-05-10

**Authors:** Sven Dittrich, Erika Graf, Regina Trollmann, Ulrich Neudorf, Ulrike Schara, Antje Heilmann, Maja von der Hagen, Brigitte Stiller, Janbernd Kirschner, Robert Dalla Pozza, Wolfgang Müller-Felber, Katja Weiss, Katja von Au, Markus Khalil, Reinald Motz, Christoph Korenke, Martina Lange, Ekkehard Wilichowski, Joseph Pattathu, Friedrich Ebinger, Nicola Wiechmann, Rolf Schröder, on behalf of the German Competence Network for Congenital Heart Defects and the Treat-NMD Neuromuscular Network Investigators list of additional local Investigators and co-workers of the German Competence Network for Congenital Heart Defects and the Treat-NMD Neuromuscular Network, Julia Halbfass, Jasmin Webinger, Anja Weise, Franz Herrndobler, Mateja Nerad, Amira Shabaiek, Güler Akin-Erdinc, Verena Greim, Dorothée Böcker, Stefanie Siepe, Sabine Schneider-Fuchs, Brigitte Egenhofer-Kummert, Barbara Burkhardt, Elena Neumann, Rudolf Korinthenberg, Christian Apitz, Matthias Freund, Michael Schumacher, Verena Gravenhorst, Daniela Deppe, Joachim Eichhorn

**Affiliations:** 1 Department Pediatric Cardiology, Erlangen University Hospital, Friedrich-Alexander Universität Erlangen-Nürnberg, Loschgestraße 15, 91054 Erlangen, Germany; 2 Institute of Medical Biometry and Statistics, Clinical Trials Unit, Faculty of Medicine and Medical Center, University of Freiburg, Freiburg, Germany; 3 Department of Pediatrics, Division of Pediatric Neurology, Erlangen University Hospital, Friedrich-Alexander Universität Erlangen-Nürnberg, Erlangen, Germany; 4 Clinic for Pediatrics III, University Hospital Essen, Essen, Germany; 5 Department of Neuropediatrics, University Hospital Essen, Essen, Germany; 6 Department of Pediatrics, University Hospital Carl Gustav Carus, Dresden, Germany; 7 Department of Neurological Surgery, University Hospital Carl-Gustav-Carus, Technical University of Dresden, Dresden, Germany; 8 Department of Congenital Heart Disease and Pediatric Cardiology, University Heart Center Freiburg, Bad Krozingen, Freiburg, Germany; 9 Department of Neuropediatrics and Muscle Disorders, University Medical Center, Freiburg, Germany; 10 Department of Pediatric Cardiology, Ludwig Maximilians-University of Munich, Munich, Germany; 11 Department of Pediatric Neurology and Developmental Medicine, Ludwig-Maximilians- University of Munich, Munich, Germany; 12 Pediatric Cardiology and Congenital Heart Disease, University Hospital Charité, Berlin, Germany; 13 Department of Pediatrics, Division of Neurology, University Hospital Charité, Berlin, Germany; 14 Division of Pediatric Heart Surgery, Pediatric Heart Center, University Hospital UKGM, Justus-Liebig University, Giessen, Germany; 15 Department of Pediatric Cardiology, Elisabeth Children’s Hospital, Oldenburg, Germany; 16 Department of Pediatric Neurology, Oldenburg, Germany; 17 Department of Pediatric Cardiology and Intensive Care Medicine, Heart Center, University Medical Center Göttingen, Göttingen, Germany; 18 Department of Pediatrics and Adolescent Medicine, Division of Pediatric Neurology, University Medical Center Göttingen, Göttingen, Germany; 19 Department of Pediatric Cardiology, University of Heidelberg, Heidelberg, Germany; 20 Pediatric Neurology, University of Heidelberg, Heidelberg, Germany; 21 Clinical Trials Unit of the Medical Center, University of Freiburg, Freiburg, Germany; 22 Institute of Neuropathology, Erlangen University Hospital, Erlangen, Germany; 23 German Competence Network for Congenital Heart Defects partner site, Berlin, Germany

**Keywords:** Duchenne muscular dystrophy, Cardiomyopathy, ACE-inhibitors, ß-blockers

## Abstract

**Background:**

X-linked Duchenne muscular dystrophy (DMD), the most frequent human hereditary skeletal muscle myopathy, inevitably leads to progressive dilated cardiomyopathy. We assessed the effect and safety of a combined treatment with the ACE-inhibitor enalapril and the β-blocker metoprolol in a German cohort of infantile and juvenile DMD patients with preserved left ventricular function.

**Methods Trial design:**

Sixteen weeks single-arm open run-in therapy with enalapril and metoprolol followed by a two-arm 1:1 randomized double-blind placebo-controlled treatment in a multicenter setting. *Inclusion criteria:* DMD boys aged 10–14 years with left ventricular fractional shortening [LV-FS] ≥ 30% in echocardiography. *Primary endpoint:* time from randomization to first occurrence of LV-FS < 28%. *Secondary:* changes of a) LV-FS from baseline, b) blood pressure, c), heart rate and autonomic function in ECG and Holter-ECG, e) cardiac biomarkers and neurohumeral serum parameters, f) quality of life, and g) adverse events.

**Results:**

From 3/2010 to 12/2013, 38 patients from 10 sites were centrally randomized after run-in, with 21 patients continuing enalapril and metoprolol medication and 17 patients receiving placebo. Until end of study 12/2015, LV-FS < 28% was reached in 6/21 versus 7/17 patients. Cox regression adjusted for LV-FS after run-in showed a statistically non-significant benefit for medication over placebo (hazard ratio: 0.38; 95% confidence interval: 0.12 to 1.22; *p* = 0.10). Analysis of secondary outcome measures revealed a time-dependent deterioration of LV-FS with no statistically significant differences between the two study arms. Blood pressure, maximal heart rate and mean-NN values were significantly lower at the end of open run-in treatment compared to baseline. Outcome analysis 19 months after randomization displayed significantly lower maximum heart rate and higher noradrenalin and renin values in the intervention group. No difference between treatments was seen for quality of life. As a single, yet important adverse event, the reversible deterioration of walking abilities of one DMD patient during the run-in period was observed.

**Conclusions:**

Our analysis of enalapril and metoprolol treatment in DMD patients with preserved left ventricular function is suggestive to delay the progression of the intrinsic cardiomyopathy to left ventricular failure, but did not reach statistical significance, probably due to insufficient sample size.

**Clinical trial registration:**

DRKS-number 00000115, EudraCT-number 2009–009871-36.

**Electronic supplementary material:**

The online version of this article (10.1186/s13023-019-1066-9) contains supplementary material, which is available to authorized users.

## Background

Mutations of the human dystrophin gene on chromosome Xp21 cause Duchenne muscular dystrophy (DMD) [1], which is the most frequently occurring muscular dystrophy in humans with an incidence of 1 in 3600–6000 male births [2]. In addition to early onset and progressive muscular weakness and wasting, which inevitably leads to loss of ambulation of boys between 9 and 13 years of age [3], nearly all DMD patients develop dilated cardiomyopathy with impaired systolic function in their second decade of life [4–8]. Although promising therapeutic options such as ataluren for stop codon read-through are available for eligible (< 10%) of the patients [9], to date, no curative therapy is available for DMD. Though multidisciplinary care, comprising early treatment with corticosteroids, physiotherapy, early antibiotic treatment of pulmonary chest infections, scoliosis surgery with insertion of spinal rods, implementation of respiratory support and drug treatment of heart failure, has substantially improved life expectancy and quality of life for DMD patients, most patients die in the second to the fourth decade of life due to combined respiratory and cardiac failure [2, 4, 10, 11]. Thus, regular cardiological and pulmonary diagnostic work-up of all DMD patients is mandatory to assess individual heart and respiratory function and to adapt therapeutic strategies [12].

In general, the medical treatment of cardiomyopathy in pediatric patients is still an open debate [13]. While evidence based studies and guidelines providing treatment recommendations for adult cardiomyopathy with impaired left ventricular function, including the use of the angiotensin converting enzyme inhibitor enalapril and the beta receptor blocker metoprolol [14, 15] exists, corresponding data for pediatric patients is vastly lacking. Thus, the rationale for the use of most heart failure medications in pediatric patients is mostly extrapolated from studies in adult heart failure [16]. In the context of DMD a number of open studies indicated that ACE inhibitors, angiotensin receptor blockers, beta-blockers and/or aldosterone antagonists might improve or preserve left ventricular systolic function and may delay the progression of cardiomyopathy [4, 17–21]. Moreover, one study demonstrated that the early intervention with perindopril led to a significantly higher overall survival in DMD patients with preserved left ventricular ejection fraction at baseline [18]. Though the comparison and interpretation of the later studies is generally hampered by their individual methodological design and the use of different outcome measurements [19], the available data supports the use of heart failure medication in DMD patients but provides no conclusive evidence regarding the optimal timing of therapy initiation [4, 19, 21, 22].

In the present multicenter study we assessed the effects of a combined therapy of the angiotensin converting enzyme inhibitor enalapril and the β-receptor blocker metoprolol on the onset of significant left ventricular dysfunction in 10–14 year old DMD boys with preserved left ventricular function.

## Methods

### Patients

Patients for this investigator-initiated, double-blind, randomized, placebo-controlled multicenter study were recruited at 10 German study sites (Berlin, Dresden, Erlangen, Essen, Freiburg, Giessen, Göttingen, Heidelberg, Munich, Oldenburg) from March, 2010 to December, 2013. Inclusion criteria for boys suffering from Duchenne muscular dystrophy were: 1) the diagnosis was based on a genetically confirmed disease causing mutation or report of negative dystrophin immunostaining in a diagnostic muscle biopsy, 2) age of 10 to 14 years, 3) preserved left ventricular function as defined by echocardiography with left ventricular fractional shortening ≥30% in the long-axis motion-mode, 4) normal renal function with glomerular filtration rate > 30 ml/min/1.73m^2^, and 5) ability to participate in the assessment of primary and secondary outcome measures. Exclusion criteria were i) any contraindication for treatment with angiotensin converting enzyme inhibitors or β-blockers, ii) previous treatment with those drugs in the past three months, iii) abnormal liver function defined by elevation (≥2x) of gamma-glutamyltranspeptidase and bilirubine, iv) left ventricular dilation above the 97th percentile as defined by echocardiography in the long-axis motion-mode, and v) participation in other clinical trials. This clinical trial was approved by the regulatory authorities and ethics committees at each study site and performed in accordance with good clinical practice guidelines. The objectives, study design, risks, and benefits of participation were explained to all participants, and written informed consent was obtained from patients and parents before enrolment.

### Open run-in, randomization and masking

The principle of anti-congestive medications requires up-titration of dosages to the individually maximum tolerated level within a safety range [14, 15]. To define the individual drug tolerance in all of the patients screened for eligibility in this study, we opted for a preceding 16 weeks open run-in period with enalapril (enalapril-maleat) and metoprolol (metoprolol-succinat). Drug dosages of enalapril and metoprolol were increased step-by-step in 3 weight classes in 4 timely shifted steps for each of the drugs up to the maximum final daily dosage of 10 mg enalapril / 47.5 mg metoprolol (patient weight < 45 kg), 10 mg enalapril / 71.25 mg metoprolol (patient weight 45 - < 60 kg) and 20 mg enalapril / 95 mg metoprolol for patients with a body weight > 60 kg. After 16 weeks open run-in period, patients were randomly assigned at a 1:1 ratio to receive either the combination of enalapril and metoprolol without interruption or placebo with a 4 weeks stepwise wash-out protocol to disguise potential rebound effects in the placebo group. A stratified block randomization with randomly varying block sizes of two or six participants and stratification for trial site was used. Allocation of patients was performed centrally by the pharmacy of the University Hospital Erlangen based on computer-generated lists. Both active drugs and placebo were supplied by Hexal AG (Holzkirchen, Germany) as identically appearing tablets. Active drugs and placebo were identically prepacked to maintain the masking for the patient and investigator by the certified pharmacy of the University Hospital Erlangen according to good manufacturing practice for pharmaceuticals. Dose levels of study medication were generally kept constant but adapted to changes in body weight classes. The use of steroids or a history of the use of steroids was recorded at baseline. During the study period start of steroid therapy was not permissible but occurred in single instances. Patients who had reached the primary endpoint or the end of the study received 4 weeks of blinded wash-out medication. Thereafter, guideline-conform treatment was at the investigator’s discretion.

### Outcome measures

The primary outcome was the time from randomization to the first occurrence of a left ventricular fractional shortening < 28% in the long-axis motion-mode of echocardiography. Corresponding analyses were performed biannually at the individual study sites. Visits continued to end of study after the primary endpoint was reached.

Secondary outcome measurements were 1) echocardiographic changes of left ventricular fractional shortening from the end of the run-in period, 2) echocardiographic changes of left ventricular diastolic diameter and systolic ventricular septum thickness measurements by motion-mode, 3) echocardiographic tissue-Doppler analyses (see below), 4) blood pressure values, 5) electrocardiograms and Holter-electrocardiograms (see below), 6) laboratory tests (see below), 7) quality of life rating (see below), and 8) adverse events.

Tissue Doppler data comprised assessment of septal, left ventricular and right ventricular longitudinal function by analysis of systolic strain in the basal, mid and apical region, respectively. Recording of tissue-Doppler data was restricted to the availability of a GE-echo-machine at the study site. All echocardiographic and tissue-Doppler data were collected in a standardized way in four-chamber-view as established by the German competence network for Congenital Heart Disease (http://www.kinderkardiologie.org/fileadmin/user_upload/Stellungnahmen/QualitaetsstandardsEcho.pdf). Tissue Doppler data were centrally analyzed by the same investigator in the tissue Doppler reference center of the German competence network for Congenital Heart Disease in Freiburg.

Electrocardiograms and Holter-electrocardiograms were centrally analyzed by a blinded investigator in Erlangen. Holter-ECG analyses included heart frequency analyses and heart rate variability measures (mean NN: average normal R to R interval; SDNN: Standard deviation of R to R intervals; SDANN: Standard deviation of the means for each R to R segment; ASDNN: average standard deviation of all 5-min R to R- intervals; rMSSD: Root-mean-Square of successive differences of NN [normal R to R intervals]; pNN50: fraction of NN intervals that differ by more than 50 ms from the previous NN interval).

Laboratory tests comprised the neurohumoral markers renin, angiotensin II, aldosterone and norepinephrine and the biomarker NT-pro-BNP.

The German Kiddo-KINDL questionnaire for adolescents aged 12–16 years [23] was used as a generic quality of life rating measure. According to the study protocol, quality of life-questionnaire was first requested at the screening visit. A complete survey of all patients was repeated one year after randomization and then annually.

The safety of enalapril and metoprolol administration was monitored from the run-in period until 30 days after discontinuation of the study drugs by adverse event reports and biannual physical examination, assessment of blood pressure, and local safety laboratory tests (including creatinine, potassium, sodium, urea, glutamate oxalacetate transaminase [GOT], glutamate pyruvate transaminase [GPT], γ-glutamyl transpeptidase [γ-GT] and bilirubin). As serum creatinine titer is not a reliable biomarker for renal function in patients with Duchenne muscular dystrophy because of their low muscle mass [24], cystatin C was measured when creatinine titers were elevated. Safety laboratory values were directly assessed by local investigators. Abnormal values considered to yield clinical significance were reported as adverse events.

### Statistical analysis

Initially, the target was 130 patients randomized within three years, plus three years additional follow-up, due to feasibility constraints. We anticipated that 50% of patients on placebo would suffer from an LV-FS < 28% after 4 years of individual follow-up^7^. With a cumulative drop-out rate of 5% up to year 4.5 (median follow-up time), a log-rank test with two-sided significance level 5% of time from randomization to first occurrence of LV-FS < 28% would have 80% power if the hazard ratio for enalapril and metoprolol versus placebo was 0.46 (Lakatos approximation, 58 events required), corresponding to an improvement to 72.7% free of left-ventricular dysfunction (LV-FS < 28%) after 4 years. Given previous results [17], a hazard ratio of 0.46 seemed achievable, but smaller treatment benefits would also be clinically relevant. Due to difficulties in recruitment, the target number was reduced to 55 patients in December 2012. This would still yield 80% power to detect a difference between treatments with respect to change in LV-FS from end of run-in to the visit scheduled 19 months after randomization (visit 4), which was considered the most relevant secondary outcome. Assuming a standard deviation of 4% at visit 4 [17], a t-test with two-sided significance level 5% would achieve this power if the mean difference 19 months after randomization was 3.1%. By December 2013, 42 patients had given informed consent, and it was decided to stop recruitment and continue follow-up until end of December 2015.

The analysis of treatment effects was done by intention-to-treat in all 38 patients who were randomized after the run-in period. In the primary analysis, time from randomization to first occurrence of an LV-FS < 28% was analyzed with the proportional hazards model, censoring at the last visit for those patients in whom no LV-FS < 28% was observed. The treatment effect was tested using the Wald-test at two-sided significance level of 5%, and was estimated as a hazard ratio with two-sided 95% confidence interval. Due to the insufficient recruitment, covariate adjustment for study site originally planned in the study protocol was replaced by adjustment for LV-FS measured after run-in in the statistical analysis plan before the blind was broken. A planned sensitivity analysis to explore a possible confounding effect of concomitant treatment with steroids was done by additional inclusion treatment with steroids as a time-dependent covariate in the primary proportional hazards model.

Secondary efficacy outcomes were analyzed in a mixed model for repeated measures including outcomes after randomization and 19 months later as endpoints and outcome after run-in, treatment, and the interaction between measurement time and treatment as covariates; subjects were modelled as random effects. Linear regression originally planned in the protocol was replaced by this longitudinal model in the statistical analysis plan to allow inclusion of all randomized patients under a missing at random assumption even if they dropped out after randomization. Changes from screening to end of run-in were summarized by means with 95% confidence intervals. Entries to the KINDL questionnaires were evaluated in accordance with the corresponding manual. Adverse events were coded by the Medical dictionary for regulatory activities (MedDRA version 19.1) and summarized single-armed (verum) for those events with onset from run-in to four weeks after randomization, two-armed (verum versus placebo) for those events with onset after that, restricting the analysis sets to those patients who received at least one dose of study medication in the corresponding period.

All *p*-values were two-sided and considered exploratory except for the primary analysis, programming was done with SAS (version 9.2) in UNIX. An independent data monitoring committee reviewed safety data on a yearly basis. An interim analysis of efficacy data, which had been planned initially, was cancelled because of the reduced target number of patients.

## Results

### Study population

**Fig. 1:**
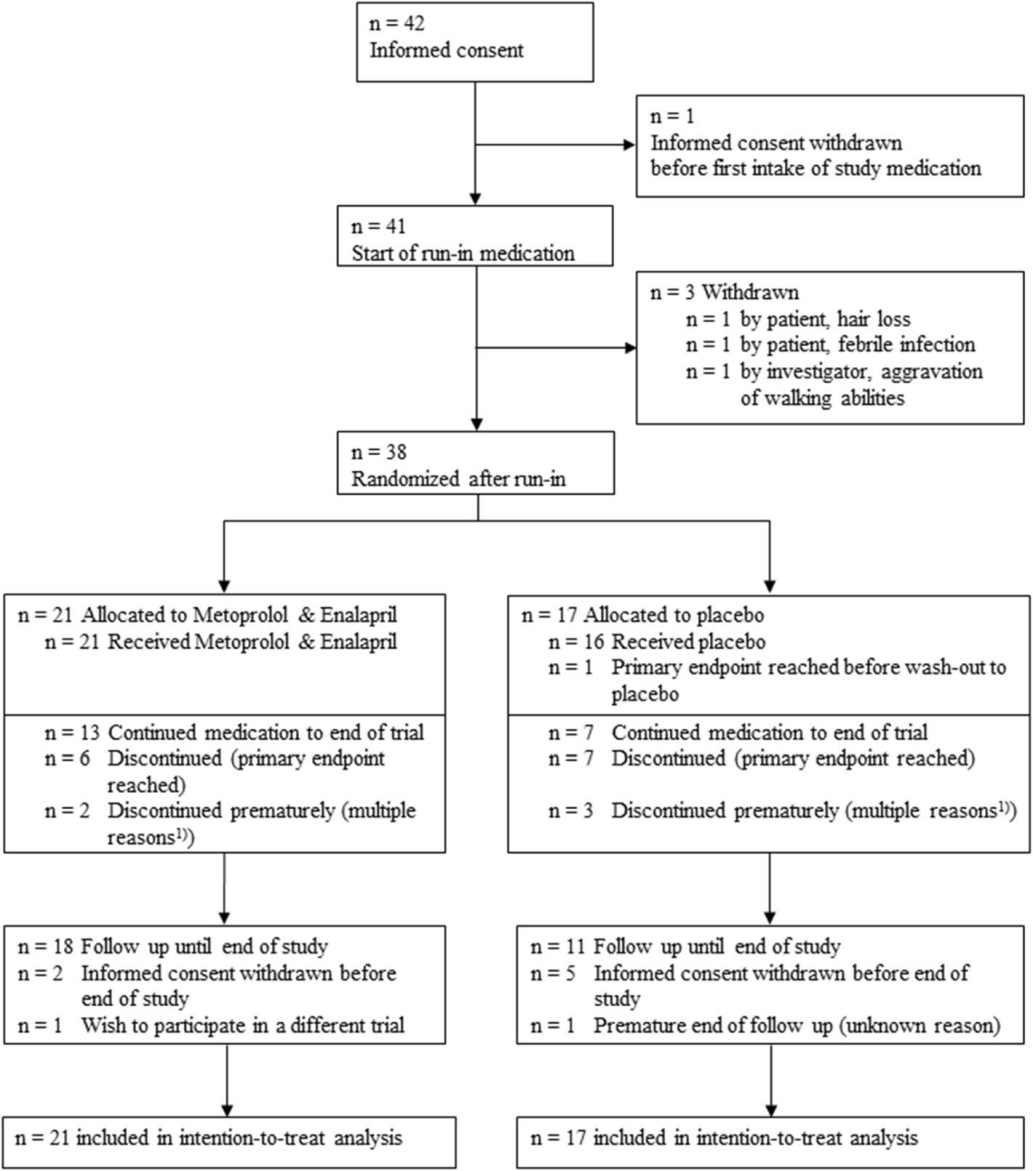
CONSORT Diagram. ^1)^ In 2 versus 3 patients (Enalapril & Metoprolol versus Placebo), intake terminated prematurely (multiple reasons could apply): 5x patient wish (2 versus 3), 3x withdrawal of consent (1 versus 2), 1x patient non-compliance (1 versus 0), and one adverse event (0 versus 1: loss of appetite, increased feeling of thirst)

Between March 2010 and December 2013, 42 boys gave informed consent, 41 started open run-in medication and 38 patients were randomized after a run-in (Fig. 1). The study was concluded with the last patient visit in December 2015.

### Outcome after open run-in phase (all patients)

During the open run-in medication period, two protocol deviations were noted: in one patient the run-in had to be repeated (due to a bone fracture) and was delayed in a second patient. Three of 41 patients dropped out of the study due to discontinuation of study medication: one patient withdrew consent because of increased hair loss, a second patient after an episode of febrile infection, nausea and vomiting, and in a third patient the local investigator stopped the medication because of decreasing walking abilities which completely recovered after disposing of the drugs. In the remaining 38 patients the maximum dose level was tolerated in 29 patients (76%), whereas 9 patients (24%) tolerated only reduced medication levels (Table 1).

**Table 1:** Outcomes before and after run-in medication (all patients)

	Screening^1)^		End of run-in^1)^		Change from screening to end of run-in^2)^	
Dose level after run-in						
maximum dose	–	–	76%	29/38	–	–
step 3 dose	–	–	18%	7/38	–	–
step 2 dose	–	–	5%	2/38	–	–
Systolic blood pressure [mmHg]	111 ± 13	*n* = 41	102 ± 14	*n* = 38	−9 [−13 to −5]*	*n* = 37
Echocardiography						
Left ventricle fractional shortening [%]	35 ± 3	n = 42	36 ± 4	n = 38	0 [−1 to 2]	*n* = 38
Electrocardiogram (ECG)						
Ventricular heart rate [beats/min]	97 ± 14	n = 41	88 ± 16	n = 38	−9 [−13 to −4]*	n = 37
Holter-Electrocardiogram (Holter-ECG)						
Ventricular heart rate [beats/min]:						
minimum	73 ± 10	n = 38	69 ± 11	n = 35	−2 [−6 to 2]	*n* = 34
maximum	140 ± 15	n = 38	129 ± 15	n = 35	−11 [−16 to −6]*	n = 34
mean	101 ± 13	n = 38	93 ± 11	n = 35	−8 [−13 to −3]*	n = 34
Heart rate variability (Holter ECG)						
mean NN [ms]	574 ± 122	*n* = 23	627 ± 140	*n* = 21	52 [24 to 81]*	*n* = 19
SDNN [ms]	85 ± 23	*n* = 22	97 ± 29	n = 21	12 [3 to 21]*	n = 19
SDANN [ms]	69 ± 24	n = 23	73 ± 24	n = 21	0 [−12 to 13]	*n* = 20
ASDNN [ms]	45 ± 18	n = 22	57 ± 21	n = 21	11 [5 to 18]*	n = 19
rMSSD [ms]	35 ± 18	*n* = 24	46 ± 23	n = 22	9 [−1 to 18]	n = 21
pNN50 [%]	9 ± 8	n = 24	15 ± 13	n = 21	5 [1 to 10]*	n = 20

* difference is statistically significant

^1)^ Data are %, x/n or mean ± SD, n

^2)^ Data are mean change [95% confidence interval], n

We observed statistically significant changes with a drop of systolic blood pressure, a shortening of QTc-time (ECG), a drop of heart rate (ECG and Holter-ECG) and of heart rate variability (Holter-ECG) (Table 1, and Additional file 1: Table S1A). All patients displayed sinus rhythm. Changes in ECG pattern indicating a right ventricular hypertrophy occurred in 1 out of 38 patients during run-in. Short episodes of ventricular tachycardia were documented in 2 out of 38 patients at screening, but were not found in any patient under medication (Additional file 1: Table S1A).

The observed changes of left ventricular fractional shortening were marginal and without statistical significance: 35 ± 4% (mean ± SD) at screening and 36 ± 4% (mean change 0.4, 95% CI -1.1 to 1.9, *p* = 0.58) in the 38 randomized patients after up-titration of the drugs (Table 1). There were no statistically significant changes in other echocardiographic measurements and in Tissue Doppler analysis (Additional file 1: Table S1A).

Changes of safety laboratory testings were marginal (Additional file 1: Table S1A) and none of the safety laboratory testings was reported as an adverse event (AE).

According to the study protocol, quality of life-questionnaire was first requested at the screening visit and complete survey of all patients was repeated one year after randomization. The overall quality of life score was 73.5 ± 10.0 (*n* = 42) and 73.3 ± 11.3 (*n* = 35), respectively.

Adverse events (AEs) with onset from run-in to four weeks after randomization were reported in 37 out of 41 patients (90%) and are listed according to MedDRA® preferred terms in Table 2 only if more than one event of the same kind was documented. Incidence of AE reports was 0.7 per person-month (142 AEs/201 person-months). One AE (muscular weakness) induced stop of medication.

**Table 2:** Incidence of adverse events with onset from start of run-in medication to 4 weeks after randomization (all patients)

Preferred term	No.	%	95% confidence intervals
Total number of patients	41	100%;	
Patients with at least one AE	37	90%	(77–97%)
Headache	11	27%	(14–43%)
Nasopharyngitis	11	27%	(14–43%)
Cough	8	20%	(9–35%)
Nausea	8	20%	(9–35%)
Febrile infection	6	15%	(6–29%)
Diarrhoea	5	12%	(4–26%)
Dizziness	4	10%	(3–23%)
Fall	3	7%	(2–20%)
Fatigue	3	7%	(2–20%)
Pyrexia	3	7%	(2–20%)
Abdominal pain	2	5%	(0.6–17%)
Back pain	2	5%	(0.6–17%)
Chest pain	2	5%	(0.6–17%)
Decreased appetite	2	5%	(0.6–17%)
Muscular weakness	2	5%	(0.6–17%)
Rash	2	5%	(0.6–17%)

Data are number of patients, percentage; 95% confidence interval

### Baseline measurements before randomization

After run-in, 38 patients were randomized across 10 sites (Fig. 1). 21 were randomly assigned to continue active medication at the dose level achieved during run-in (enalapril and metoprolol). 17 patients were assigned to receive placebo after a four weeks blinded wash-out phase (placebo). Baseline characteristics of the patients by randomized treatment are given in Table 3. At the point of randomization, baseline heart rate (ECG and Holter-ECG) as well as heart rat variability values such as mean NN were unequally distributed among the enalapril and metoprolol and the placebo group. Patients randomized to placebo treatment had higher heart rates and larger mean NN-values (Table 3).

**Table 3:** Baseline characteristics by randomized treatment (end of run-in therapy)

	Enalapril and Metoprolol ^1)^		Placebo ^1)^	
Age [years]	12 ± 1.2	n = 21	11 ± 1.1	*n* = 17
Body Mass Index [kg/m^2^]	23 ± 6	n = 20	21 ± 5	n = 17
Ability to rise from supine position	24%	5/21	24%	4/17
Preserved ability to walk	33%	7/21	41%	5/17
Maximum walking distance [m]	200 (3–6000)^2)^	*n* = 7	300 (30–800)^2)^	*n* = 5
Systolic blood pressure [mmHg]	103 ± 16	n = 21	101 ± 11	n = 17
Patients with presence of cardiac symptoms	0%	0/21	0%	0/17
Steroid use or history of steroid use	76%	16/21	59%	10/17
NYHA class				
Not applicable	52%	11/21	59%	10/17
NYHA class I	48%	10/21	35%	6/17
NYHA class II	0%	0/21	6%	1/17
Quality of life (KINDL total score at screening)	70.8 ± 10.1	n = 21	75.6 ± 10.0	n = 17
Echocardiography		n = 21		n = 17
Left ventricle fractional shortening [%]	35 ± 3		36 ± 4	
Electrocardiogram (ECG)		n = 21		n = 17
Ventricular heart rate [beats/min]	84 ± 14		94 ± 16	
Holter-Electrocardiogram (Holter-ECG)		n = 20		*n* = 15
Ventricular heart rate [beats/min]				
minimum	67 ± 12		72 ± 10	
maximum	126 ± 17		133 ± 11	
mean	90 ± 12		97 ± 9	
Heart rate variability (Holter ECG)		*n* = 11		*n* = 10
Mean NN [ms]	678 ± 80		571 ± 174	
SDNN [ms]	98 ± 29		96 ± 30	
SDANN [ms]	71 ± 24		75 ± 25	
ASDNN [ms]	60 ± 20		54 ± 21	
rMSSD [ms]	46 ± 26		45 ± 21	
pNN50 [%]	17 ± 15		13 ± 11	

^1)^ Data are mean ± SD or percentage, n = number of measurements

^2)^ Data are mean, (minimum-maximum), n = number of measurements

Demographic data were collected at screening, baseline data from echocardiography, ECG and Holter-ECG refer to measurements after run-in/before randomization

### Outcome after randomization

Patient follow-up for the primary endpoint included 108 person-years, and study visits took place until end of study in 29 of 38 patients. Three versus 6 patients (Enalapril and metoprolol versus placebo) discontinued study visits prematurely, thereof 1 versus 3 patients after they had reached the primary endpoint (Fig. 1).

### Results-efficacy-primary

**Fig. 2:**
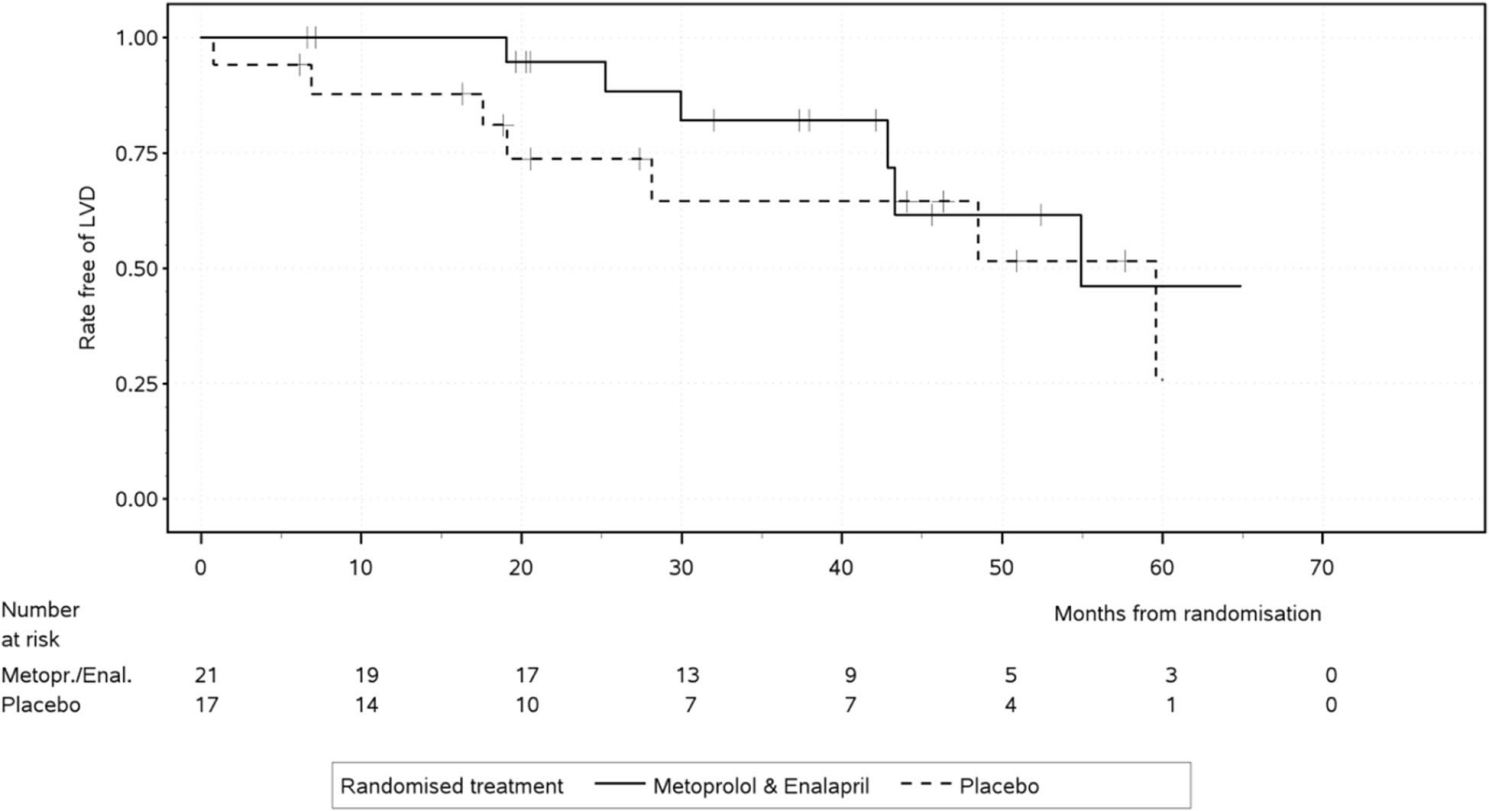
Kaplan-Meier plot for time to left ventricular fractional shortening < 28%. Enalapril and metoprolol compared to placebo seemed to be in favour for left ventricular shortening < 28% for the first three years (n.s.). After 3.5 years, the estimated rates of patients free of left-ventricular dysfunction in treated and non-treated patients converged. Abbreviations: LVD = left ventricular dysfunction

After randomization, a LV-FS < 28% was observed in 6 of 21 and 7 of 17 patients assigned to Enalapril and Metoprolol versus placebo, respectively. For the primary endpoint, time from randomization to the first occurrence of LV-FS < 28%, Cox regression adjusted for LV-FS after run-in showed a statistically non-significant benefit for enalapril and metoprolol over placebo (hazard ratio [HR] 0.38; 95% confidence interval [CI] 0.12 to 1.22; *p* = 0.10) (Fig. 2).

Left ventricular fractional shortening after run-in had a significant impact on time to left ventricular fractional shortening < 28%: Each percent point after run-in lowered the hazard of left ventricular dysfunction by a factor (HR) of 0.72 (95%CI 0.55 to 0.93, *p* = 0.011).

Concomitant steroid treatment was given at least once after randomization in 10 of 21 patients on enalapril and metoprolol versus 11 of 17 patients on placebo. Sensitivity analysis to investigate a potential confounding impact by inclusion of a time-dependent indicator of steroid intake did not alter the estimated effect of enalapril and metoprolol versus placebo (HR 0.32; 95% CI 0.09 to 1.13; *p* = 0.076). The effect of steroid intake on time to first occurrence of LV-FS < 28% was estimated as a HR of 0.61 (95%CI 0.16 to 2.37; *p* = 0.47).

### Results-efficacy-secondary

Change of left ventricular fractional shortening was considered the most relevant secondary efficacy endpoint. The difference between treatments at month 19, estimated as 0.62% in favor of enalapril and metoprolol (Table 4), was not statistically significant (95%CI − 1.98 to 3.22%, *p* = 0.63). Adjusted analysis for LV-FS after run-in showed that LV-FS decreased by − 0.10% per month in the enalapril and metoprolol -group (95%CI − 0.21 to 0.02%, *p* = 0.10) compared to − 0.13% per month with placebo (95%CI − 0.25 to 0.00%, *p* = 0.042). We observed no effect on left ventricular diameter or ventricular thickness (Table 4).

**Table 4:** Outcome at 19 months after randomization

	Enalapril and Metoprolol		Placebo		Adjusted Difference ^1)^	
Systolic blood pressure [mmHg]	104 ± 13	n = 19	108 ± 14	*n* = 14	−2.5 [−10.9 to 5.9]	n = 38
Echocardiography						
Left ventricle fractional shortening [%]	34 ± 3.9	n = 19	33 ± 5.7	n = 15	0.6 [−2.0 to 3.2]	n = 38
Left ventricle diastolic diameter [cm]	4 ± 0.5	n = 19	4 ± 0.4	n = 15	0.1 [−0.2 to 0.4]	n = 38
Interventricular septum systolic thickness [cm]	1 ± 0.2	*n* = 12	1 ± 0.5	*n* = 8	−0.1 [−1.5 to 1.3]	*n* = 18
Electrocardiogram (ECG)						
Ventricular heart rate [beats/min]	84 ± 16	n = 18	96 ± 12	n = 15	−4.8 [−12.5 to 2.9]	n = 38
P-wave [ms]	84 ± 14	n = 18	73 ± 14	n = 15	10.3 [2.1 to 18.6]*	n = 38
PQ-interval [ms]	129 ± 20	n = 18	115 ± 12	n = 15	10.9 [2.1 to 19.7]*	n = 38
QRS-time [ms]	102 ± 67	n = 18	86 ± 9	n = 15	5.5 [−2.4 to 13.3]	n = 38
QTc-time [ms]	405 ± 28	n = 18	415 ± 33	n = 15	−9.2 [−26.4 to 8.0]	n = 38
Holter-Electrocardiogram (Holter-ECG)						
Ventricular heart rate [beats/min]:						
Minimum	68 ± 12	n = 15	75 ± 12	n = 14	−2.6 [−10.5 to 5.3]	*n* = 32
Maximum	123 ± 14	n = 15	136 ± 15	n = 14	−16.7[−25.6 to −7.9]*	n = 32
Mean	91 ± 13	n = 15	101 ± 14	n = 14	−5.2 [−12.8 to 2.3]	n = 32
Heart rate variability (Holter ECG)						
mean NN [ms]	681 ± 86	n = 8	650 ± 82	*n* = 6	−54.1 [− 160.2 to 52.0]	n = 17
SDNN [ms]	85 ± 31	*n* = 9	90 ± 46	n = 7	−17.8 [−52.4 to 16.9]	n = 18
SDANN [ms]	59 ± 29	n = 9	66 ± 36	n = 7	−19.0 [−47.4 to 9.4]	n = 18
ASDNN [ms]	52 ± 18	n = 9	55 ± 33	n = 7	−13.0 [−37.5 to 11.6]	n = 18
rMSSD [ms]	37 ± 15	n = 9	41 ± 32	n = 7	−12.9 [−40.2 to 14.3]	n = 19
pNN50 [%]	12 ± 9	n = 9	13 ± 14	n = 7	−6.8 [−20.3 to 6.7]	n = 18
Quality of life (KINDL total score) ^2)^	74.7 ± 12.3	n = 15	76.4 ± 6.4	n = 14	1.5 [−4.3 to 7.4]	*n* = 36
Biomarker and neurohumoral markers ^3)^						
NT-proBNP [pg/ml]	90 ± 69	*n* = 16	52 ± 42	*n* = 13	12 [− 13 to 38]	*n* = 28
Noradrenalin [pg/ml]	355 ± 139	n = 12	188 ± 58	n = 9	124 [21 to 228]*	n = 17
Renin [pg/ml]	297 ± 357	n = 16	38 ± 43	n = 12	332 [119 to 545]*	*n* = 27
Aldosteron [ng/ml]	0.077 ± 0.097	n = 13	0.056 ± 0.036	n = 11	0.032 [−0.027 to 0.090]	n = 24
Angiotensin II [pmol/ml]	19.4 ± 32.4	n = 16	15.6 ± 13.8	n = 12	−6.1 [−31.3 to 19.1]	n = 27

Data are mean ± SD, n = number of measurements

* difference is statistically significant

^1)^ Differences adjusted for baseline measurements after run-in; information from subjects with missing values at month 19 included in mixed model for repeated measures

^2)^ Differences adjusted for KINDL-questionnaire at screening; information from subjects with missing values at month 19 included in mixed model for repeated measures

^3)^ Measurements were taken at a mean of 19 months (12.1 to 26.2 months). Differences adjusted for measurements at screening

Adjusted differences between treatments were not statistically significant for systolic blood pressure (Table 4).

All patients had sinus rhythm during whole study period. No episodes of supraventricular or ventricular tachycardias had been registered in any Holter-ECG recordings.

Baseline distribution of heart frequencies after run-in in ECG and Holter-ECG was asymmetric (Table 3). Adjusted differences showed significantly lower maximal ventricular heart rate in Holter-ECG in the enalapril and metoprolol group compared to placebo (Table 4).

Changes of heart rate variability parameters were statistically significant as analyzed for all patients during open run-in medication for an increase of meanNN, an increase of SDNN, an increase of ASDNN and an increase of pNN50 (Table 1). The values were asymmetrically distributed at randomization baseline (Table 3). Adjusted differences between randomized treatments after 19 months were not significant (Table 4).

NT-pro-BNP values were within a low range at screening (see Additional file 1: Table S2A) and after 19 months of randomized treatment (Table 4). This also applies for values of the renin–angiotensin–aldosterone system (RAAS) (Table 4, Additional file 1: Table S2A). However, we observed significant adjusted differences with an increase of noradrenalin and renin values in the enalapril and metoprolol group (Table 4).

The KINDL total quality of life score did not deteriorate with time and showed no difference between treatments at month 19 (Table 4). Pooled data for subscales are visualized in the Additional file 1: Fig. S1A).

### Results-safety/tolerability

After randomization, the majority of patients (33 of 38) continued intake of study medication either up to the end of the trial (14 versus 7, enalapril and metoprolol versus placebo) or until the primary endpoint was reached (Fig. 2). In 2 versus 3 patients, intake terminated prematurely. Reasons (multiple reasons could apply) included 5x patient wish (2 versus 3), 3x withdrawal of consent (1 versus 2), 1x patient non-compliance (1 versus 0) and one adverse event (0 versus 1: loss of appetite, increased feeling of thirst). We noticed 13 protocol deviations: Adaptation of dose level to increased body weight was delayed in 11 patients (4 enalapril and metoprolol, 7 placebo), not done in one patient and prematurely done in another patient (both enalapril and metoprolol). No unblinding occurred.

Adverse events (AEs) with onset after randomization and the four weeks wash-out period of the placebo arm were reported in 21/21 versus 15/16 (enalapril and metoprolol versus placebo) of the patients. Table 5 shows AEs that were documented in more than one patient per arm. Incidence of AE reports was 0.24 versus 0.26 per person-month on study medication (enalapril and metoprolol: 181 AEs/739 person-months, placebo: 129 AEs/490 person-months). The total number of patients with at least one serious AE (SAE) was 8/21 versus 7/16. None of the SAEs was clearly related to verum or placebo medication. One patient in the placebo-group stopped drug intake prematurely due to increased hair loss (compare to hair loss, which led to withdrawal in 1 patient during run-in-period).

**Table 5:** Incidence of adverse events with onset 4 weeks after randomization by received treatment

	Enalapril and Metoprolol		Placebo		Difference between groups	
Preferred term	No.	(%)	No.	(%)	(%)	95% confidence intervals
Total number of patients	21	(100%)	16	(100%)		
Patients with at least one AE	21	(100%)	15	(94%)	6%	(−10 to 28%)
Febrile infection	11	(52%)	5	(31%)	21%	(−10 to 47%)
Nasopharyngitis	10	(48%)	5	(31%)	16%	(−15 to 43%)
Diarrhoea	6	(29%)	1	(6%)	22%	(−4 to 44%)
Cough	4	(19%)	5	(31%)	−12%	(−39 to 15%)
Headache	4	(19%)	5	(31%)	−12%	(− 39 to 15%)
Mechanical ventilation	3	(14%)	1	(6%)	8%	(−16 to 29%)
Abdominal pain upper	3	(14%)	0		14%	(−7 to 35%)
Gastroenteritis	3	(14%)	0		14%	(−7 to 35%)
Spinal operation	2	(10%)	3	(19%)	−9%	(−34 to 14%)
Immunisation	2	(10%)	2	(13%)	−3%	(−27 to 18%)
Pyrexia	2	(10%)	2	(13%)	−3%	(−27 to 18%)
Lower limb fracture	2	(10%)	1	(6%)	3%	(−20 to 23%)
Oropharyngeal pain	2	(10%)	1	(6%)	3%	(−20 to 23%)
Chest pain	2	(10%)	0		10%	(−11 to 29%)
Fatigue	2	(10%)	0		10%	(−11 to 29%)
Humerus fracture	2	(10%)	0		10%	(−11 to 29%)
Influenza like illness	2	(10%)	0		10%	(−11 to 29%)
Photosensitivity reaction	2	(10%)	0		10%	(− 11 to 29%)
Tonsillitis	2	(10%)	0		10%	(−11 to 29%)
Upper respiratory tract infection	2	(10%)	0		10%	(−11 to 29%)

Data are number of patients, percentage; difference between groups (%), 95% confidence intervals of difference (%)

## Discussion

This randomized, double-blinded and placebo-controlled trial investigated the effect of a combined ACE-inhibitor and beta-blocker treatment on the progression to DMD-related cardiomyopathy in boys with preserved left ventricular function and between 10 and 14 years of age. As the primary endpoint of this study, the time from randomization to the first occurrence of LV-FS < 28% in the long-axis motion-mode of echocardiography was chosen. The obtained results indicate a slower progression to left ventricular failure in DMD patients of this age group receiving this combined pharamacological intervention. Notably, the observed HR of 0.38 was even more in favour of enalapril and metoprolol than anticipated at planning (0.46), and substantially more patients were free of left-ventricular dysfunction for the first three years (Fig. 2). However, these results did not reach statistical significance, presumably due to the insufficient sample size. After 3.5 years, the estimated rates of patients free of left-ventricular dysfunction in treated and non-treated patients converged (Fig. 2). This might be a random effect of the small remaining number of patients at risk after 3.5 years (5 vs. 4 patients, Fig. 2). The here reported beneficial effects of enalapril and metoprolol over placebo should be interpreted in view of the fact that all patients started the study with medication of enalapril and metoprolol in the run-in-period (Fig. 1), which might have had a persistent effect in the placebo group [18] and thus lowered the outcome differences between the two treatment groups.

Though direct comparison of our results with other work addressing the effects of ACE-inhibitor and / or beta-blockers treatment in the context of DMD cardiomyopathy are intrinsically hampered by differences in the individual study design (i.e. applied inclusion criteria for case selection, specific medication, chosen diagnostic workup), further studies support the notion of the here reported beneficial effects. Mono-therapy with enalapril in a 2-year follow-up randomized trial with 21 patients with 42 DMD or BMD patients (mean age 12.1 years) with preserved left ventricular function was reported to decelerate the progression of myocardial fibrosis as quantified by CMR [21]. Eplerenone, an aldosterone antagonist, which was used in combination with an ACE inhibitor or an angiotensin receptor blocker, was reported to elicit a slight deceleration of left ventricular circumferential strain decline assessed by CMR in a 12 month follow-up period in 20 DMD patients with preserved left ventricular function (mean age 14.5 years). Here, the median decline of left ventricular circumferential strain was 1% in the active treatment group versus 2.2% in the placebo group [4]. The aforementioned reduced decline of left ventricular circumferential strain by eplerenone in combination with an ACE inhibitor or an angiotensin receptor blocker treatment was further confirmed in 11 DMD patients in a 2-years open-label extension trial [22]. Three further studies implicated that the use of ACE inhibitor or eplerenone treatment may attenuate, but not prevent, the deterioration of LV systolic function [4, 17, 21, 25, 26], which is typically observed in DMD cardiomyopathy [4, 17, 21, 25, 26]. With regard to improvement of survival of DMD patients, two studies outlined positive effects by the early initiation of an ACE-inhibitor in patients with preserved left ventricular function [17, 18]. Moreover, ACE-inhibitor plus β-blocker treatment was reported to be more beneficial in patients with asymptomatic compared to those with symptomatic heart failure [27], and the combination therapy with an ACE-inhibitor or angiotensin receptor blocker plus β-blocker compared to mono-therapy was more favorable in DMD patients with abnormal left ventricular ejection fraction [28].

In line with earlier studies [21, 22, 26], we observed a relatively slow decline of global left ventricular function in our series of DMD patients. Here, our analysis showed that left ventricular fractional shortening decreased by − 0.10% per month in the enalapril and metoprolol group compared to − 0.13% per month in the placebo group (95%CI − 0.25 to 0.00%, *p* = 0.042).

In our study up-titration of enalapril and metoprolol without concealment was performed to test individual tolerance of the guideline recommended high dosages for anti-congestive indication [29]. The results of this run-in period show that boys with DMD very well tolerate effective doses of medication with regard to blood pressure, which in general is low in DMD patients. Drop of blood pressure did not lead to withdrawals or adverse event reporting in our series of patients. High heart rates due to autonomous nerve system impairment have previously been reported in DMD patients [30–32] and were also observed in the current study. During open run-in treatment with ACE inhibitors and beta-blockers we observed the expected effects on heart rate and ECG and heart frequency variability [32]. However, these did not show any obvious impact on left ventricular measurements by echocardiography.

In the present study, special emphasis was further put on the observation of safety, side effects and compliance of the possibly life-long medication in patients, whose quality of life already is severely hindered by severe muscular dystrophy. While our analysis revealed a relatively good compliance, neither meaningful differences of adverse effects nor a negative impact on the quality of life became apparent in the comparison between treatment groups.

## Conclusions

Our analysis of initiation of a combined therapy with the ACE-inhibitor enalapril and the β-blocker metoprolol in DMD patients younger than 14 years of age and with preserved left ventricular function is suggestive to delay the progression of the intrinsic cardiomyopathy to left ventricular failure. However, this delay did not reach statistical significance, probably due to an insufficient sample size. In our patients long-term treatment with this combination therapy was safe and well tolerated, and no negative impact on quality of life was seen.

## Additional file

Additional file 1: **Table S1A.** Outcomes before and after run-in medication (all patients, additional measurements). **Table S2A.** Baseline characteristics by randomized treatment (additional measurements). **Figure S1A.** Results from KINDL-questionnaire. (DOCX 72 kb)

## Abbreviations

ACE: Angiotensin converting enzyme
AE: Adverse event
ASDNN: Average standard deviation of all 5-min R to R- interval
DMD: Duchenne muscular dystrophy
ECG: Electrocardiogram
LV-FS: Left ventricular fractional shortening
mean NN: Average normal R to R interval
NN: R to R interval
NT-pro-BNP: N-terminales pro brain natriuretic peptide
pNN50: Fraction of NN intervals that differ by more than 50 ms from the previous NN interval SDANN Standard deviation of the means for each R to R segment
rMSSD: Root-mean-Square of successive differences of NN
SDNN: Standard deviation of R to R intervals
