# Supplementary material for: Effect and safety of treatment with ACE-inhibitor Enalapril and β-blocker metoprolol on the onset of left ventricular dysfunction in Duchenne muscular dystrophy - a randomized, double-blind, placebo-controlled trial
Source: Orphanet J Rare Dis. 2019 May 10;14:105. doi: 10.1186/s13023-019-1066-9 (PMC6509833; doi:10.1186/s13023-019-1066-9)
Supplement: Supplementary file 1 — Table S1A. Outcomes before and after run-in medication (all patients, additional measurements). Table S2A. Baseline characteristics by randomized treatment (additional measurements). Figure S1A. Results from KINDL-questionnaire. (DOCX 72 kb) [file 13023_2019_1066_MOESM1_ESM.docx]

# Appendix: Supplemental material

**Table 1A Outcomes before and after run-in medication (all patients, additional measurements)**

|  | Screening^1)^ | |  | End of run-in^1)^ | |  | Change from screening to end of run-in^2)^ | |
| --- | --- | --- | --- | --- | --- | --- | --- | --- |
| **Safety laboratory testings** |  |  |  |  |  |  |  |  |
| - Potassium [mmol/l] | 4.1±0.4 | n=42 |  | 4.3±0.5 | n=37 |  | 0.2 [0.1 to 0.4]* | n=37 |
| - Sodium [mmol/l] | 140±2 | n=42 |  | 140±3 | n=37 |  | -0.4 [-1.2 to 0.4] | n=37 |
| - Creatinin [mg/dl] | 0.23±0.13 | n=42 |  | 0.22±0.11 | n=37 |  | -0.01 [-0.03 to 0.01] | n=37 |
| - Urea [mg/dl] | 19±6 | n=42 |  | 22±7 | n=37 |  | 2 [0 to 3]* | n=37 |
| - Glutamate oxalacetate transaminase (GOT) [U/l] | 113±51 | n=42 |  | 104±56 | n=37 |  | -8 [-18 to 2] | n=37 |
| - Glutamate pyruvate transaminase (GPT) [U/l] | 179±103 | n=42 |  | 165±98 | n=37 |  | -17 [-28 to -6]* | n=37 |
| - γ-glutamyl transpeptidase (γ-GT) [U/l] | 16±13 | n=42 |  | 17±12 | n=37 |  | 0.4 [-2.5 to 3.3] | n=37 |
| - Bilirubin [mg/dl] | 0.4±0.2 | n=42 |  | 0.4±0.2 | n=36 |  | -0.04 [-0.10 to 0.01] | n=36 |
| - Cystatin C [mg/l] | 0.69±0.12 | n=38 |  | 0.72±0.12 | n=37 |  | 0.02 [-0.01 to 0.06] | n=33 |
| **Echocardiography** | | | | | | | | |
| - Left ventricle diastolic diameter [cm] | 4.0±0.5 | n=42 |  | 4.0±0.5 | n=38 |  | 0.0 [-0.1 to 0.1] | n=38 |
| - Interventricuar septum systolic thickness [cm] | 1.3±0.3 | n=18 |  | 1.4±0.4 | n=18 |  | 0.0 [-0.2 to 0.3] | n=14 |
| **Tissue Doppler (systolic strain in four-chamber view)** | | | | | | | | |
| - Interventricuar septum IVS-Basal [%] | -19±5.2 | n=16 |  | -19±7.5 | n=12 |  | 1.4 [-3.9 to 6.7] | n=12 |
| - Interventricuar septum IVS-mid [%] | -17±4.5 | n=15 |  | -18±7.2 | n=11 |  | -1.0 [-7.2 to 5.2] | n=11 |
| - Interventricuar septum IVS-apical [%] | -11±6.7 | n=15 |  | -12±6.5 | n=12 |  | -1.3 [-5.2 to 2.5] | n=12 |
| - Left ventricular wall LV-basal [%] | -19±7.5 | n=16 |  | -13±15.6 | n=12 |  | 5.6 [-4.4 to 15.5] | n=12 |
| - Left ventricular wall LV-mid [%] | -17±9.6 | n=15 |  | -19±9.3 | n=11 |  | -1.6 [-6.4 to 3.2] | n=10 |
| - Left ventricular wall LV-apical [%] | -13±5.0 | n=12 |  | -13±8.8 | n=10 |  | 0.4 [-5.9 to 6.7] | n=10 |
| - Right ventricular wall RV-basal [%] | -36±8.0 | n=12 |  | -34±16.2 | n=11 |  | 4.1 [-4.5 to 12.7] | n=10 |
| - Right ventricular wall RV-mid [%] | -26±15.0 | n=10 |  | -28±11.6 | n=9 |  | -2.5 [-14.2 to 9.2] | n=7 |
| - Right ventricular wall RV-apical [%] | -15±6.8 | n=9 |  | -18±6.7 | n=8 |  | -2.3 [-9.8 to 5.3] | n=5 |

| **Electrocardiogram (ECG)** | | | | | | | | |
| --- | --- | --- | --- | --- | --- | --- | --- | --- |
| - P-wave [ms] | 75±12 | n=41 |  | 75±10 | n=38 |  | 0 [-5 to 5] | n=37 |
| - PQ-interval [ms] | 118±14 | n=41 |  | 119±15 | n=38 |  | 1 [-3 to 6] | n=37 |
| - QRS-time [ms] | 82±9 | n=41 |  | 80±10 | n=38 |  | -2 [-7 to 2] | n=37 |
| - QTc-time [ms] | 414±21 | n=41 |  | 404±24 | n=38 |  | -13 [-22 to -3]* | n=37 |
| - Pattern of right ventricular hypertrophy | 0% | 0/41 |  | 2.6% | 1/38 |  | 2.6% | 1/38 |
| - Pattern of left ventricular hypertrophy | 0% | 0/41 |  | 0% | 0/38 |  | 0% | 0/38 |
| **Holter-Electrocardiogram (Holter-ECG)** | | | | | | | | |
| - Presence of supraventricular tachycardia | 0% | 0/38 |  | 0% | 0/35 |  | 0% | 0/34 |
| - Presence of ventricular tachycardia | 5.3% | 2/38 |  | 0% | 0/35 |  | 0% | 0/34 |
| * difference is statistically significant  ^1)^ Data are %, x/n or mean±SD, n.  ^2)^ Data are mean change [95% confidence interval], n. | | | | | | | | |

Table 1A complements table 1 and provides detailed data of all additional measurements which have been performed according to the study protocol. The presentation comprises all patients prior and independent of randomization at week 16 to demonstrate possible effects of run-in medication. Analysis of safety laboratory testings at screening and after 16 weeks of combined enalapril and metoprolol medication after deblinding the study showed a little increase of potassium, a little increase of urea and a little decrease of glutamate pyruvate transaminase (GPT) measurements, which as typical finding in DMD patients have been slightly elevated at screening. No significant effects of treatment were observed on left ventricular diameter and interventricular septal thickness. Tissue Doppler systolic longitudinal strain was analyzed in a subgroup of patients. Values showed the typical distribution pattern with a regular decrease in strain from the base to the apex of the septum. Changes after treatment were not significant.

**Table 2A Baseline characteristics by randomized treatment (additional measurements)**

|  | **Metoprolol & Enalapril** | | **Placebo** | |
| --- | --- | --- | --- | --- |
| **Biomarker and neurohumoral markers (at screening)** |  |  |  |  |
| - NT-proBNP [pg/ml] | 54±37 | n=20 | 51±55 | n=17 |
| - Noradrenalin [pg/ml] | 266±108 | n=17 | 241±109 | n=15 |
| - Renin [pg/ml] | 17±10 | n=20 | 22±8 | n=17 |
| - Aldosteron [ng/ml] | 0.079±0.075 | n=20 | 0.093±0.050 | n=16 |
| - Angiotensin II [pmol/ml] | 6.5±3.5 | n=19 | 10.0±3.6 | n=16 |
| **Echocardiography** |  |  |  |  |
| - Left ventricle diastolic diameter [cm] | 4.0±0.4 | n=21 | 4.0±0.5 | n=17 |
| - Interventricular septum systolic thickness IVS [cm] | 1.3±0.3 | n=11 | 1.5±0.5 | n=9 |
| **Electrocardiogram (ECG)** |  | n=21 |  | n=17 |
| - Ventricular heart rate [beats/min] | 84±14 |  | 94±16 |  |
| - P-wave [ms] | 76±9 |  | 75±10 |  |
| - PQ-interval [ms] | 121±15 |  | 116±13 |  |
| - QRS-time [ms] | 80±12 |  | 79±7 |  |
| - QTc-time [ms] | 403±23 |  | 406±24 |  |
| - Pattern of right ventricular hypertrophy | 5% | 1/21 | 0% | 0/17 |
| - Pattern of left ventricular hypertrophy | 0% | 0/21 | 0% | 0/21 |
| **Holter-Electrocardiogram (Holter-ECG)** |  | n=20 |  | n=15 |
| - Mean ventricular heart rate [beats/min] | 90±12 |  | 97±9 |  |
| - Presence of supraventricular tachycardia | 0% | 0/20 | 0% | 15/15 |
| - Presence of ventricular tachycardia | 0% | 0/20 | 0% | 15/15 |

Table 2A complements table 3 with additional measurements.

Data are mean±SD or percentage, n=number of measurements.

Neurohumoral markers were collected at screening, baseline data from echocardiography, ECG and Holter-ECG refer to measurements after run-in/before randomization.

**Figure 1A Results from KINDL-questionnaire**

Within a time period of 5 years from screening, the self-reported QOL did not change. Scales remain within the same range and show comparable allocation of mean values over the time indicating that patients life standard did not improve and deteriorate neither. This finding is remarkable in regard of the progression of DMD (loss of ambulatory abilities at this age) Even the scale of body remained almost at the same level.

Compared to healthy boys in the normal population, the scales body and friends are lower in the study population. This images the limitations of patients, who have to sit in a wheelchair and cannot meet with friends as easily as healthy teens. The scales self, family and especially school are higher in the study population compared to normal population. This illustrates the special meaning of family for the patients and may show a high commitment to perform in school. However, as a limitation to this interpretation, it should be noted that comparisons to reference values may not be reliable since patients in our trial were aged 10 to 14 years at inclusion. For the sake of consistency and comparability, the Kiddo KINDL questionnaire valid at the start of the trial for children of age 12 to 16 years was applied throughout the study. Lateron, the editors changed the applicable age range of the identical questionnaire to 14 to 17 years. The evaluation manual remained unchanged.

**^*^ list of additional local Investigators and co-workers of the German Competence Network for Congenital Heart Defects and the Treat-NMD Neuromuscular Network**

- Julia Halbfass, Department Pediatric Cardiology, Erlangen University Hospital, Friedrich-Alexander Universität Erlangen-Nürnberg, Erlangen, Germany
- Jasmin Webinger, Department Pediatric Cardiology, Erlangen University Hospital, Friedrich-Alexander Universität Erlangen-Nürnberg, Erlangen, Germany
- Anja Weise, Department Pediatric Cardiology, Erlangen University Hospital, Friedrich-Alexander Universität Erlangen-Nürnberg, Erlangen, Germany
- Franz Herrndobler, Department Pediatric Cardiology, Erlangen University Hospital, Friedrich-Alexander Universität Erlangen-Nürnberg, Erlangen, Germany
- Mateja Nerad, Department Pediatric Cardiology, Erlangen University Hospital, Friedrich-Alexander Universität Erlangen-Nürnberg, Erlangen, Germany
- Amira Shabaiek, Department Pediatric Cardiology, Erlangen University Hospital, Friedrich-Alexander Universität Erlangen-Nürnberg, Erlangen, Germany
- Güler Akin-Erdinc, Department Pediatric Cardiology, Erlangen University Hospital, Friedrich-Alexander Universität Erlangen-Nürnberg, Erlangen, Germany
- Verena Greim, Department Pediatric Cardiology, Erlangen University Hospital, Friedrich-Alexander Universität Erlangen-Nürnberg, Erlangen, Germany
- Dorothée Böcker, Department Pediatric Cardiology, Erlangen University Hospital, Friedrich-Alexander Universität Erlangen-Nürnberg, Erlangen, Germany
- Stefanie Siepe, Clinical Trials Unit of the Medical Center, University of Freiburg, Freiburg, Germany
- Sabine Schneider-Fuchs, Clinical Trials Unit of the Medical Center, University of Freiburg, Freiburg, Germany
- Brigitte Egenhofer-Kummert, Clinical Trials Unit of the Medical Center, University of Freiburg, Freiburg, Germany
- Barbara Burkhardt, University Heart Center Freiburg-Bad Krozingen, Department of Congenital Heart Disease and Pediatric Cardiology, Medical Center-University of Freiburg, Faculty of Medicine, University of Freiburg, Freiburg, Germany
- Elena Neumann, Department of Congenital Heart Defects and Pediatric Cardiology, Heart Centre, University of Freiburg, Freiburg, Germany
- Rudolf Korinthenberg, Department of Neuropediatrics and Muscle Disorders, Medical Center, University of Freiburg, Freiburg, Germany
- Christian Apitz, Pediatric Heart Center, University Hospital UKGM, Justus-Liebig University , Division of Pediatric Heart Surgery , Giessen, Germany
- Matthias Freund, Department of Paediatric Cardiology, Elisabeth Children's Hospital, Oldenburg, Germany
- **Michael Schumacher,** Department of Paediatric Cardiology, Elisabeth Children's Hospital, Oldenburg, Germany
- Verena Gravenhorst, Department of Paediatric Cardiology and Intensive Care Medicine, Heart Center, University Medical Center Göttingen, Göttingen, Germany
- Daniela Deppe, Department of Paediatric Cardiology, University Medical Center Göttingen, Göttingen, Germany
- Joachim Eichhorn, Department of Paediatric Cardiology, University of Heidelberg, Heidelberg, Germany
